# Exploring sociodemographic disparities in diagnostic problems and mistakes in the quest for diagnostic equity: insights from a national survey of patient experiences

**DOI:** 10.3389/fpubh.2025.1444005

**Published:** 2025-02-13

**Authors:** Kathryn M. McDonald, Kelly T. Gleason, Rachel N. Grob, Christina T. Yuan, Isha Dhingra, Jane A. Evered, Emily M. Warne, Mark Schlesinger

**Affiliations:** ^1^School of Nursing, Johns Hopkins University, Baltimore, MD, United States; ^2^School of Medicine, Johns Hopkins University, Baltimore, MD, United States; ^3^Qualitative and Health Experiences Research Lab, Department of Family Medicine and Community Health, University of Wisconsin-Madison, Madison, WI, United States; ^4^School of Public Health, Johns Hopkins Bloomberg, Baltimore, MD, United States; ^5^School of Public Health, Yale University, New Haven, CT, United States

**Keywords:** patient experience, diagnostic equity, population-based survey, patient safety, diagnostic errors, household survey methodology, sociodemographic risk factors

## Abstract

**Introduction:**

As part of building a platform for epidemiological research on diagnostic errors and problems that centers on patients and equity, this paper summarizes the development and analysis of data collected from fielding a survey in a nationally representative U.S. population to explore the prevalence and harm consequences of diagnostic problems or mistakes (referred to here as “diagnostic P&Ms”) by respondent-reported sociodemographic characteristics.

**Methods:**

We applied narrative elicitation methods to enhance the rigor of implementing a novel survey about diagnostic experiences. We conducted a U.S. population-based survey of a nationally representative sample in 2022–2023, drawn from the NORC AmeriSpeak® panel. We conducted multivariate regression analysis at the household level and in a patient subsample to explore sociodemographic predictors of diagnostic P&Ms and related outcomes in the aftermath.

**Results:**

The comparative analysis by sociodemographic characteristics estimates prevalence of diagnostic P&Ms, prevalence of persisting harms, rate of respondent-reported perceptions of personal attribute adversely affecting diagnosis, and concern about future diagnostic P&Ms. Outcome estimates ranged from about 4% (concern about future diagnostic P&M) to 38% (at least one P&M in households during the past 4 years). Several sociodemographic groups experienced statistically significant higher levels of risk for these outcomes, with some at greater than twice the odds compared to reference groups—transgender and gender independent individuals (e.g., 5 + −fold odds of expectation of future P&M compared to cis-males), cis-females (e.g., greater than 1.5 odds of persistent physical and emotional harms compared to cis-males), low household income (e.g., twice the likelihood of multiple P&Ms for incomes under $60 K compared to $100 K+ households), younger age (3-fold odds of at least one diagnostic P&M for those under 25 years old compared to those aged 45–54), multiracial individuals (about twice the odds of diagnostic P&Ms compared to non-Hispanic White), and disabled and unable to work full-time (more than twice the likelihood of perceiving that a personal attribute impaired diagnosis compared to those with other work status designations).

**Discussion:**

This new survey and accompanying data source facilitate an enriched exploration of the patterns of diagnostic disparities and points of leverage through which diagnostic experiences can be made more equitable.

## 1 Introduction

Diagnostic errors pose significant risks to public health, contributing to adverse patient outcomes and systemic inefficiencies (1). Despite growing recognition of their impact (2, 3), and new studies documenting the aggregate scope of diagnostic errors in the United States (4), there remains a notable paucity of studies quantifying how the risk of diagnostic errors varies among different population subgroups (5). To date, the research demonstrating the heightened vulnerability for some populations is largely based on data from particular practice sites, practice settings, or health conditions (6–8). Because methods are inconsistent across these studies, the evidence on diagnostic inequities remains fragmented and inadequate in several ways.

First and foremost, most extant studies identifying diagnostic inequities do so using clinical markers, rather than patients’ own assessments. Because key aspects of diagnosis—including the effectiveness of clinicians’ communication and their responsiveness to patient-reported symptoms, concerns, and experiences—can *only* be reliably assessed through patients’ reports, many potential manifestations of diagnostic inequity remain underexamined (5, 9, 10). Moreover, when patients perceive that some personal attribute has deleteriously affected their diagnosis, the potential damage to their relationships with clinicians and/or the wider health care system can lead to persisting loss of trust in medical care, increased concern about misdiagnosis in the future, and weakening of the therapeutic alliance between patients and clinicians essential for accurate and timely diagnosis (11–13).

Second, the handful of published studies that do include patient-reported diagnostic errors and problems (14–17) have relied on relatively small samples. This makes it difficult to sort out which attributes of combined sociodemographic constructs, such as lower socio-economic status (e.g., limited education and limited income reported together in one variable) represent the real predictors of the identified inequities. Because past studies have identified a number of intercorrelated sociodemographic characteristics such as age, gender and sex, race and ethnicity, disability, and economic status—each of which has been individually associated with elevated diagnostic risks—the intersections and interactions of attributes have not, to date, been effectively parsed out (7, 8, 14, 18).

Finally, because the evidence-base currently documenting diagnostic inequities is aggregated from a set of narrowly focused studies, it has been impossible to reliably compare the magnitude of diagnostic shortfalls or harms across different subgroups. This undermines efforts to prioritize among interventions that might reduce diagnostic inequities, because they cannot be sensibly targeted to the groups experiencing the greatest current burdens.

To address these gaps in our understanding of diagnostic disparities and inequities, we developed a novel survey specifically designed for learning more about patients’ and household care partners’ assessments of diagnostic experiences. Our survey, developed and fielded in a national panel, provides the first ever household-reported data set for comprehensive analysis of “diagnostic problems and/or mistakes” (abbreviated as “diagnostic P&Ms”) in the United States to reflect the lived experience of patients and their household care partners. These “diagnostic P&Ms” refer to any problem and/or mistake identified by patients themselves or the people living with them (hereafter referred to as household care partners). They include diagnostic P&M events that “can be caused by not getting enough information from the patient, not ordering the right tests in a timely way, not reading test results correctly, or doctors not sharing information well enough with one another.” (See Exhibit 1.) The broader focus on P&Ms is intended to better align survey responses with elucidating safety threats and informing actions in the quest for diagnostic excellence inclusive of diagnostic equity. Although some diagnostic P&Ms may not equate to clinically adjudicated diagnostic errors, they represent lived experiences with problems and mistakes in the public’s experience of diagnosis that can undergo epidemiologic analysis. With a large sample –almost 4,000 households screened to identify 1,500+ events reported as P&Ms related to diagnosis—we can better distinguish among correlated attributes associated with elevated risks or harm.

**Exhibit 1:**
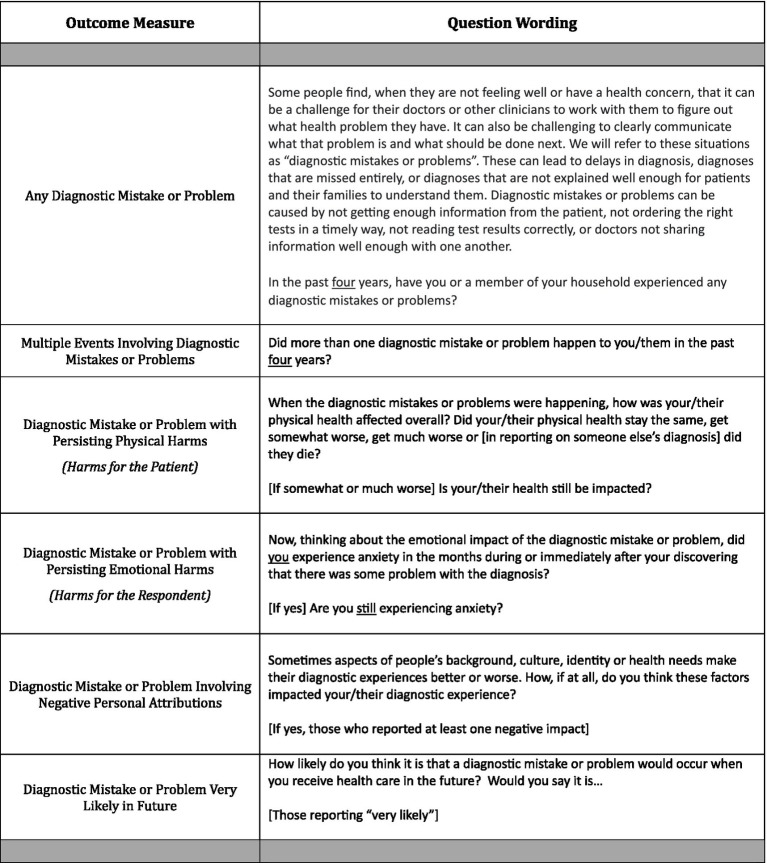
Survey question wording for outcome variables.

The analysis presented here assesses the sociodemographic correlates of diagnostic P&Ms at both the household and patient level. In this paper, we present the methodology used to conduct our survey, describe the characteristics of the study population, and analyze the sociodemographic predictors of diagnostic P&Ms along with their subsequent effects on patients. We report on the prevalence of diagnostic P&Ms and persisting harms by sociodemographic factors such as household income, gender identity, age, marital status, education, race and ethnicity, disability work status and urban/rural residence. We also estimate how these same personal attributes are related to respondents’ expectations regarding future diagnostic risks.

Understanding the distribution of diagnostic disparities is crucial to inform development of targeted interventions to reduce diagnostic P&Ms and persisting harms, to surface deficiencies in diagnostic excellence (19), and ultimately to improve healthcare outcomes most equitably (5, 20, 21). Furthermore, we discuss the implications of our findings for healthcare practice, health delivery systems, policy, and directions for future research. Through our analysis, we aim to contribute valuable insights to the complex epidemiology of patient-reported diagnostic P&Ms and advance efforts towards promoting data-informed and patient-centric diagnostic equity.

## 2 Methods

The survey that generated the data for the analysis presented below was developed to provide a more robust and patient-centric representation of the diagnostic experiences of the American public. Its conceptual foundation closely accords with the one recently published by Bell and colleagues (10), though our approach (a) operationalizes an alternative way of labeling the sorts of experiences that “count” as diagnostic P&Ms, (b) embodies a commitment to rigorously eliciting narrative accounts about those diagnostic experiences, and (c) incorporates attention to patient experiences, outcomes, and expectations in the aftermath of the diagnostic P&M. We describe below the survey development process and the specific wording of key questions.

### 2.1 Source of the data

Survey data were collected from a randomly selected subset of people participating in NORC’s AmeriSpeak® online panel of over 50,000 households, designed to elicit participation from historically underrepresented populations to ensure that respondents are representative of the American public (22, 23). The panel methods used, similar to other online panels, are transparently documented and frequently assessed for reliability and representativeness (24–27). Online surveying options include two response modes: Computer-Assisted Web Interviewing (CAWI) and Computer-Assisted Telephone Interviewing (CATI). NORC collects and regularly updates information on all panelists, which makes it possible to assess the sociodemographic characteristics of respondents who screened out of the full survey because no one in their household had experienced a diagnostic P&M.

Participants in the AmeriSpeak® panel receive participation points for responding to surveys. Those participating in a survey of the length of the NEP-DE study receive compensation worth approximately $5.00.

### 2.2 Survey development

The survey used in this study was developed using a three-stage iterative process that began in April 2022 and ran through May 2023. The initial version of the survey built upon prior work on other patient safety concerns (e.g., treatment and medication errors), literature reviews, and team members’ extensive experience in survey methods and diagnostic care assessment (17, 28–31). We incorporated a rigorous narrative elicitation protocol (NEP) methodology to construct the question sequence including 10 open-ended questions that encourage a robust, balanced, and complete account from respondents (32–34, 75). It centered an inclusive understanding of patients’ and care partners’ lived experiences with the diagnostic process and outcomes, not linked to any particular care setting. The survey went through multiple phases of pilot testing, triangulation with qualitative interview data on a subset of respondents, and revisions to establish a robust and feasible set of survey questions. The novel survey is referred to as NEP-DE (see Appendix). This process was supported by input from an advisory group of patient advocates with lived experience with diagnostic P&Ms, clinicians with expertise in diagnosis and in identifying diagnostic P&Ms, and researchers with expertise in the elicitation and assessment of patient narratives.

### 2.3 Survey questions assessing outcomes

Although some previous patient experience surveys have aspired to identify events that the public views as “diagnostic errors” (35), researchers attentive to patient experience have increasingly recognized that the public views adverse diagnostic events in broader terms (36–38). Indeed, the public does not always relate to the concept of a medical error, even when it is defined for them on a survey (10). To provide a more inclusive scope, our survey asked about experiences with “mistakes and/or problems” during diagnosis (see Exhibit 1). In separate analyses of the survey, we have noted that those diagnostic P&Ms identified by patients or care partners as “problems” have, on average, as frequent and substantial harms as those that they view as “mistakes.” In other work, we have also documented that acknowledgement of something going wrong from someone in a healthcare setting occurred in about one out of three P&M reports (39).

To assess the frequency with which diagnostic P&Ms are encountered, we screened respondents aged 18+ from NORC’s AmeriSpeak® online panel regarding the diagnostic experiences of people in their households during the previous 4 years. Extended lookback periods are common for surveys of patient experiences involving safety events. Past surveys included lookback periods of 1, 4, 7 and 10 years (16, 35, 38, 40). Our use of a four-year lookback on this survey corresponds to about the midpoint of this range.

In response to the wording in the screening invitation, respondents initially identified whether anyone in their household had experienced a diagnostic P&M in the previous 4 years. Those who responded affirmatively were then asked if there had been more than one such diagnostic P&M during that time period. About half (51%) of those who reported at least one event indicated that there had been multiple events in their household. These were then prioritized by algorithm—diagnostic P&Ms that involved the respondent’s own health care were given priority, and respondents were directed to describe the most memorable P&M for themselves. Diagnostic P&Ms in which the respondent had been a household care partner were included only if the respondent had no personal experience about themselves to report. For household care partner reported events, respondents were again guided to select the most memorable P&M to further elaborate what had happened on a single P&M.

Respondents were asked multiple questions about the selected diagnostic safety event. As shown in Exhibit 1, these included whether the event had induced physical harms for the patient that still persisted at the time of the survey, emotional harms for the respondent that still persisted at the time of the survey, and whether the diagnostic experience had been negatively affected by the system or clinicians in relationship to one or more of the patient’s personal attributes. This final outcome was quantified on the basis of coding responses to the last open-ended question from the sequence of 10 questions incorporated into the NEP (see Appendix). Finally, patients were asked about their expectations regarding future diagnostic risks; responding on a four-point scale that ranged from “very likely” to “not at all likely” (Exhibit 1).

For illustrative purposes, we assembled excerpts from the open-ended questions (see Appendix) and the responses that related to selected outcomes: diagnostic P&Ms, persisting physical and emotional harms (an indicator of severe impact), and respondent perception that personal attributes impaired diagnosis. Three steps were required to provide examples of each outcome from the two perspectives: patient reports and care partner reports. First, we selected a subset of responses to represent all outcomes of interest. Second, we selected excerpts to reflect a range of writing styles and narrative lengths. Third, we crafted each excerpt using verbatim text with only minor revisions for readability (e.g., capitalizations where appropriate, but no changes to phrasing or words used), and assembling narrative segments for conciseness and continuity without necessarily reflecting the exact order or full text available in the original open-ended response.

### 2.4 Survey questions assessing sociodemographic predictors

The sociodemographic characteristics used to identify patterns of disparities in diagnostic experiences rely on information collected from all AmeriSpeak® panelists, as well as additional information collected during the survey process (Exhibit 2). Past studies relying on smaller scale or setting-specific samples suggest that certain subgroups of respondents are likely to be at heightened risk for diagnostic P&Ms, including patients from ethnic or racial minority groups (8), those with physical disabilities (18), patients from sexual and gender minority groups (41), women (7), younger and older adults (7), and those from disadvantaged socioeconomic households (14). We also include two additional sociodemographic variables that are plausibly related to so-called “upstream” determinants of diagnostic inequities (5, 42): rural residents (who face larger travel burdens in seeking out diagnoses, particularly when these involve specialists) and respondents with more limited educational attainment. Finally, we include marital status as this social factor has been shown to be protective for health outcomes in other contexts (43).

**EXHIBIT 2:**
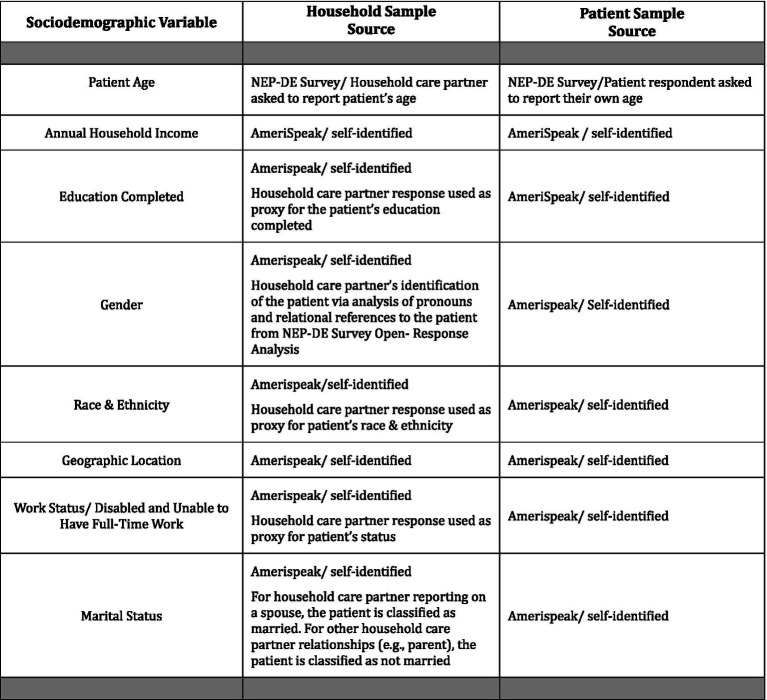
Sources for sociodemographic variables.

The terminology for sociodemographic categories (Exhibit 2) were chosen to align with NORC’s questions and response options (22). In addition, terminology for several subgroups was adapted based on additional sources (44, 45). For example, while we refer to a gender category, we use terminology for three population subcategories to be inclusive of populations who have non-binary gender identities: cis-male, cis-female, and transgender and gender independent (44). This choice aligns with the panelist responses to four choices for the question “how do you describe yourself?”—male, female, transgender or do not identify as male, female or transgender. The multiple races category refers to two or more races, and we use the term multiracial when referring to individuals in this population (45).

### 2.5 Sample selection

The AmeriSpeak® national panel was utilized to recruit participants for the survey. Panelists were offered the opportunity to complete a “survey about healthcare experiences”; 26.5% of those offered agreed to participate. Out of this participant pool, 43.6% reported having had a household member (oneself or someone else in the household) with some form of diagnostic mistake or problem during the previous 4 years. Of those who screened into the survey based on having a health care experience and then agreeing to participate, 95.4% completed the entire set of questions about adverse experiences with diagnosis.

Because the survey incorporated an extensive set of open-ended questions, which described the nature of the reported diagnostic event, we were able to further screen the reported P&M to ensure that the problems were in fact associated with diagnosis rather than treatment. Based on analysis of the narrative responses, we excluded 5.6% of the cases reported from the AmeriSpeak® respondents; these can be viewed as “false positives.”

The analytic sample was further restricted by two additional considerations. First, because income is reported at the household (shared residence) level, we excluded all P&Ms reported among family members who no longer shared the residence—this excluded a total of 270 reported P&Ms. Second, because the AmeriSpeak® panel includes only respondents 18 and older, we also excluded all cases reported by household care partners that involved patients under the age of 18—this excluded an additional 45 reported P&Ms from this analysis.

### 2.6 Data collection

We fielded the survey in three waves, the first in April of 2022, the last in May of 2023. After each of the first two waves of data collection, we further refined the question wording and sequencing. The median time to complete the survey was 23 min. Debriefing questions situated at the end of the survey suggested few difficulties in understanding or completing any of the questions.

Changes to the survey included: (a) after the first wave of the survey, altering the way in which respondents identified when they first began to search for a diagnosis in the sentinel case, so that the response included the month as well as the year of initiation, (b) altering the sequencing and/or wording of three of the prompts for the open-ended questions between the second and third waves, and (c) introducing a new question in the second wave which asked respondents to identify whether, at the time they completed the survey, the uncertainties associated with their diagnosis had been fully resolved.

The narrative elicitation sequence on this survey included 10 open-ended questions, six related to the diagnostic process, four to experiences after the respondent had determined that there had been a mistake or problem related to the diagnosis (see Appendix). The mean response time for the open-ended question sequence was 8.5 min, the median 7 min.

### 2.7 Statistical analysis

We estimated two different sets of regressions. The first set estimated the prevalence of any patient-reported diagnostic P&Ms or persistent harms. Slightly more than half (54.5%) of the cases of diagnostic P&Ms involved respondent-reported care for themselves. The second set of models estimated the prevalence of P&Ms and persistent harms from the full sample for any member of the respondent’s household, including reports from both patients and household care partners in the sample.

Multivariate regression models were estimated to identify the relationship between sociodemographic factors and diagnostic outcomes. For binary outcome variables (any P&M, multiple P&Ms, any persisting physical harms, any persisting emotional harms, any evidence that diagnosis was impaired by inappropriate attention to one or more of patient’s personal attributes) the regressions were estimated as logistic models. When the perceived future risk of a diagnostic problem was the outcome, the models were estimated as ordered logistic regression.

### 2.8 Ethical considerations

The study was deemed exempt by the IRBs at Yale (#2000032012) and Johns Hopkins (IRB00322791) universities. The study fell under the umbrella exemption granted by the University of Wisconsin IRB to qualitative projects conducted by the Qualitative and Health Experiences Research Laboratory in the Department of Family Medicine.

### 2.9 Data availability

Data from this study will be made available upon request from the corresponding author, after June 1, 2025.

## 3 Results

### 3.1 Respondent characteristics

A total of 3,995 AmeriSpeak® panelists responded to the screening questions. As shown in Table 1, the sociodemographic characteristics of these respondents mirrored those of the general U.S. adult population as of 2020 with some modest divergences.

**Table 1:** Respondent characteristics compared to general U.S. population [unweighted sample].

Respondent attributes	Source	AmeriSpeak® panelists completing screening survey	U.S. adult (18+) population, 2020	
Patient age (adults)	1			
18–24 Years Old		9.8%	12.1%	
25–34 Years Old		17.7%	15.4%	
35–44 Years Old		15.5%	18.3%	
45–54 Years Old		13.9%	15.8%	
55–64 Years Old		18.4%	16.8%	
65–74 Years Old		16.7%	12.8%	
75 Years and Older		8.0%	8.8%	
Annual household income	5			
Under $30,000		22.3%	22.1%	
$30,000–$59,999		26.5%	22.0%	
$60,000–$99,999		26.6%	22.4%	
$100,000 and Above		24.7%	33.6%	
Education completed	4			
Some High School		7.3%	13.7%	
High School Grad		19.8%	28.5%	
Some College		38.8%	28.2%	
College Grad		19.4%	11.6%	
Graduate School		14.7%	18.1%	
Gender	2,3			
Cis-Female		49.6%	50.5%	
Cis-Male		49.0%	47.2%	
Transgender and Gender Independent		1.4%	1.6–2.3%	*
Race and ethnicity	4			
White non-Hispanic (NH)		62.5%	62%	
Black NH		13.1%	12%	
Asian and Pacific Isles, NH		2.8%	6.5%	
Other Race NH		1.2%	1%	
Multiple Races NH		2.7%	2%	
Hispanic (all races)		17.7%	17%	
Geographic location	5			
Urban/Metro		84.6%	80.0%	
Rural/Non-metro		15.4%	20.0%	
Health and employment status	5			
Working or Looking for Work		67.3%	63.4%	
Not in Labor Force, Nondisabled		27.0%	29.2%	
Disabled, Unable to Have Full-time Work		5.8%	7.4%	
Marital status	1			
Married		45.5%	53.0%	
Single, Divorced, Separated, Widowed		54.5%	47.0%	

1. American Community Survey (2021). 2. Pew Foundation. 3. Census Household Pulse Survey July 21–September 13, 2021. 4. National Health Interview Survey. 5. Shrider, EA, M Kollar, F Chen, J Semega, Current Population Reports, P60-273, Income and Poverty in the United States: 2020, U.S. Government Publishing Office, Washington, DC, September 2021. *Census questions for transgender and gender independent group are still in pilot testing. Percentages show range across wording.

The respondents were slightly older with the most marked difference in the 65 and older groups (24.7% versus 21.6% in the general population). Although there were fewer of the youngest adults (18 through 24 years old) (9.8% versus 12.1%), the proportions of those under the age of 35 years old was the same for respondents versus the general population, 27.5% in each case.

Annual household income distribution was notably similar to the general population for the lowest income group of under $30,000 (22.3% versus 22.1%). There were fewer respondents reporting incomes above $100,000 compared to the general population (24.7% vs. 33.6%), and more respondents in the two middle income brackets (roughly 26% vs. 22% for each).

The educational attainment of respondents tended to be higher than the general population with more completing some college or graduating from college (58.2% vs. 39.8% combining these two categories), but fewer completing graduate school (14.7% versus 18.1%). Fewer respondents were at the low end of the educational attainment distribution (some high school or high school graduate with 27.1% versus 42.2% combined categories for respondents compared to the general population).

The distribution of gender among respondents closely aligns with that of the general population, with roughly equal proportions of cis-females and cis-males. The transgender and gender independent group is also similar to the low end of the Census estimates (1.4% versus 1.6%).

While the distribution of race and ethnicity among respondents mostly reflects that of the general population, the proportion of Asian and Pacific Islander individuals is substantially lower compared to the general population (2.8% versus 6.5%). The proportions of several other race and ethnicity groups are slightly higher among respondents compared to the general population (e.g., 17.7% versus 17% for Hispanics [all races], 13.1% versus 12% for Black, and 2.7% versus 2% for those reporting multiple races).

Although the majority of respondents reside in urban or metro areas, consistent with the distribution in the general population, there were fewer respondents from rural or non-metro locations (15.4% versus 20% in the general population). The distribution of health and employment status among respondents is largely comparable to that of the general population, with a somewhat lower proportion of individuals reporting disabled status (unable to have full-time work) among respondents compared to the general population (5.8% vs. 7.4%). The respondent sample had a smaller proportion of those currently married than did the general population, though in each case the sample was fairly evenly divided between those who were married and those not.

Overall, the comparison between respondent characteristics and the general U.S. adult population suggests that the sample captured a diverse and representative population, enhancing the generalizability of the study findings.

### 3.2 Illustrative examples of study outcomes

Table 2 presents narrative examples excerpted from patient and care partner reports to illustrate study outcome variables. Examples of diagnostic P&Ms include delays in diagnosis, problems with diagnostic testing, nonspecific diagnosis, and unresolved diagnosis. Examples of persisting physical harms reflecting severe impact of the diagnostic P&M include chronic pain, damage to extremities and nerves, and continuing functional limitations. Examples of persisting emotional harms indicative of severe impact of the diagnostic P&M include significant frustration, anger, feelings of invisibility, and stigmatization for patients reporting about themselves. Similarly, care partners experiences of persistent emotional harms include expressions such as “it broke my heart” and “it has affected the family and myself in ways words cannot express.” Examples of a personal attribute or combination of personal attributes that impaired diagnosis and suggest diagnostic inequity include gender, too young an age for the diagnosis ultimately determined, being a person with a disability, weight (“because you are fat”), and being Latina.

**Table 2:** Illustrative excerpts of outcome variables.

Outcome variable	Illustrative excerpt
Diagnostic P&M	Patient Report
	Issue with back. I thought disk was herniated but doctor refused to look into it. Was later diagnosed as a herniated disk almost 8 months later after more damage had been done. Zero treatments were effective in helping it…
	I went to the health clinic to get blood drawn to diagnose my potential thyroid problem. I do not know if it was the personnel at the clinic or the transporters that mishandled my samples, but they had to call me back to get more blood drawn because they could not get the information they needed to properly diagnose me from the first set of samples.
	Care Partner Report
	My father had a very bad rash (itching, blisters) that was first diagnosed by his PCP as just a rash. I searched on the internet and eventually came across Bullous Pemphigoid. After a second visit to the PCP, he agreed with me.
	My wife was having pain in her legs causing discomfort when walking… She did have a mass of blue veins that she thought might be the problem. She was told it was a dermatology problem. She later had some veins stripped from her legs. The problem continued. She had either two or three custom made lifts for her shoes, and then went to the “Good Feet Store” trying to get rid of the pain. In all cases, she was told that it should help. Nothing has helped, and she still has the problem which is getting worse.
Persisting Physical Harms Ò Diagnostic P&M with Severe Impact	Patient Report
	I had lots of breathing issues and chest pain at night… It was the third doctor who found the 5-inch-long tumor in my chest that was pressing on my lungs and causing pleurisy and pain… I wish I had requested imaging early on. All it took was a simple CT scan to diagnose the problem correctly, but it took almost a year and 3 doctors to get that scan. I was miserable for months and months. And after the surgery that removed the tumor, I was left with nerve damage and chronic pain that I still have that everyone tells me to just deal with. Overall, a horrible experience.
	The pain continued and worsened until belatedly doctors indicated surgery would be required. Because the situation had deteriorated so badly by then, the main nerves in my left leg became so damaged that they were no longer capable of normal use, and the operation failed to solve the problem. I now must use a walker at all times in order to stand or walk. My PCP has since admitted that an earlier surgery would have probably saved the leg.
	Care Partner Report
	[I wish I had known] that Guillan-Barre can take years to resolve and sometimes does not. What the other disabling factors are… They remained disabled with no definitive diagnosis.
	The doctors previously had been so damn preoccupied with the mysterious covid they could not be bothered with pneumonia everyone has known about and treated successfully for decades. He almost died because they ignored his lungs, his trouble breathing. All they cared about was covid… Almost 3 years later [he] is just now weaning himself off of extra oxygen to get through a normal day. He will never get back to the active life he lived before because his lungs are so damaged… Our lives will never be the same. We used to kayak when we camped. We cannot do that anymore. He does not have the strength. He cannot do a lot of the car maintenance he used to do. Very cold air, and very hot air bothers him. He just has to stop where before he could have kept doing what he was working on. It frustrates him sometimes.
Persisting Emotional Harms Ò Diagnostic P&M with Severe Impact	Patient Report
	I had chronic productive coughing, and recurring lung infections. I was diagnosed with bronchitis 3 times, walking pneumonia once (for 4 different incidents of infection). Then it became too hard to work and deal with this. I barely slept. I could not stop coughing. I was then told I had RADS—Reactive airway disorder. I then sought out information about RADS and it was clear I did NOT have that. Then I was diagnosed with whooping cough, without giving me a pertussis test… just because I had uncontrollable coughing fits. I was FINALLY referred to a pulmonologist after an ER visit due to my inability to breathe. The pulmonologist thought it was uncontrolled allergies. I had an allergy test and started immunotherapy. That helped, but I still had infections and wheezing. I was finally diagnosed with asthma last July. The treatment for asthma has really helped me… I described my situation in detail. Providers pulled answers sometimes out of their asses. Tests were not performed to try to narrow down my problems, just to support their next hypothesis. I felt invisible, unheard, and very frustrated. I was devastated emotionally. My boss tried to performance manage me out of my job because I was worried more about staying alive than her pet projects. I had to reach out to several mental health therapists to cope with my frustration. I’m gray-haired and (at the time) approaching 60, and female. I am invisible. They just want me to go away.
	I’ve been bedridden 80% of the day for around 10 years. I’m considered a chronic pain patient, which is the “unsorted” bin where they toss those of us without a clear diagnosis… I’m on Medicaid (Title 19) and the program does not pay enough to make them care. When they could not figure out what was wrong, or why, my symptoms suddenly became “all in my head.” They suggested a psychiatrist might be helpful. I suggested they should all go to hell and refused further visits. That’s about where things stand presently. When I’m forced to interact with other doctors it gets ugly really quickly.
	Care Partner Report
	My daughter had her breast removed as she is high risk for breast cancer. Nobody, including her PCP who really was now responsible for her follow up care informed her about the fact that her bone density should be taken care of. She found out about 8 months after the surgery, her bones were bones of an 80-year-old and she is only 38 years old… She Cried a Lot. A Real Lot. It broke my heart. She is doing everything in her power to strengthen her bones and it has not been easy by any means. She works on it every day and it is strenuous.
	My grandmother had fallen and was bleeding from her ears. Family knew immediately she had a concussion. Took her to our town’s local E.R. where doctors cleaned off the blood and said she did not have a concussion and sent her home. She was immediately acting strange and a couple days later [we] took her back to the E.R. because she was getting worse and that’s when the family and my grandmother found out from the Radiologist and Neurologist that she had a level 4 concussion. She never recovered properly and passed away due to complications from the concussion… It has affected the family and myself in ways words cannot express. Mentally, emotionally, and physically.
Personal Attribute(s) Perceived as Impairing Diagnosis Ò Diagnostic P&M Inequity	Patient Report
	Was misdiagnosed with an infection. I was then given incorrect medication which led to an allergic reaction… I was called names accused of being a difficult patient and because I am legally blind my competency was questioned. When I disagreed about the diagnosis and treatment the nurse questioned my competency. When I tried to involve administrators I was harassed. For a short time I refused to see any medical professional out of fear. During that time my condition worsened. I reached out to a mental health professional who encouraged me to do my own research and reach out to a new doctor… I lost the ability to walk. I had to drop out of my last semester of college. I isolated myself and was severely depressed. I refused to trust any medical professional. I ended up with irreversible joint damage. To this day I still have trust issues. Because of my experiences I only seek out care when things get really bad… I know there were factors such as my gender, being legally blind and my age that impacted my experience. Even though women are more likely to be diagnosed with an autoimmune disease we still live in the shadow of health care being designed around men. When you are younger than the typical person diagnosed you are not taken seriously. And being a person with a disability you are treated as though you cannot possibly be competent to make your own decisions. I am confident that if these factors were not present, things would have gone differently.
	I wish a doctor had taken all of my symptoms into thought instead of looking only at individual symptoms. All my symptoms taken together perfectly fit my actual diagnosis… I frequently received diagnoses that boiled down to “It’s because you are fat.” I’m very overweight and all my other symptoms were frequently ignored. In the end, weight gain was a symptom and not the problem.
	Care Partner Report
	His primary doc refused to biopsy a mass he had grown on his abdomen due to his age and the mass having characteristics of a lipoma. A year later they removed the mass due to its continued growth and it was found to be cancerous… His age impacted his diagnosis because since he was 30 they acted like it was impossible for him to have cancer.
	My wife injured herself at work and went to the hospital and they told her that she had a bruised muscle but ended up being her shoulder… They apologized and said that I was in my right to make a complain and that I did… I seen the difference in service from her being Latina and other races at the hospital.

### 3.3 Prevalence of diagnostic P&Ms and their effects

**Figure 1:**
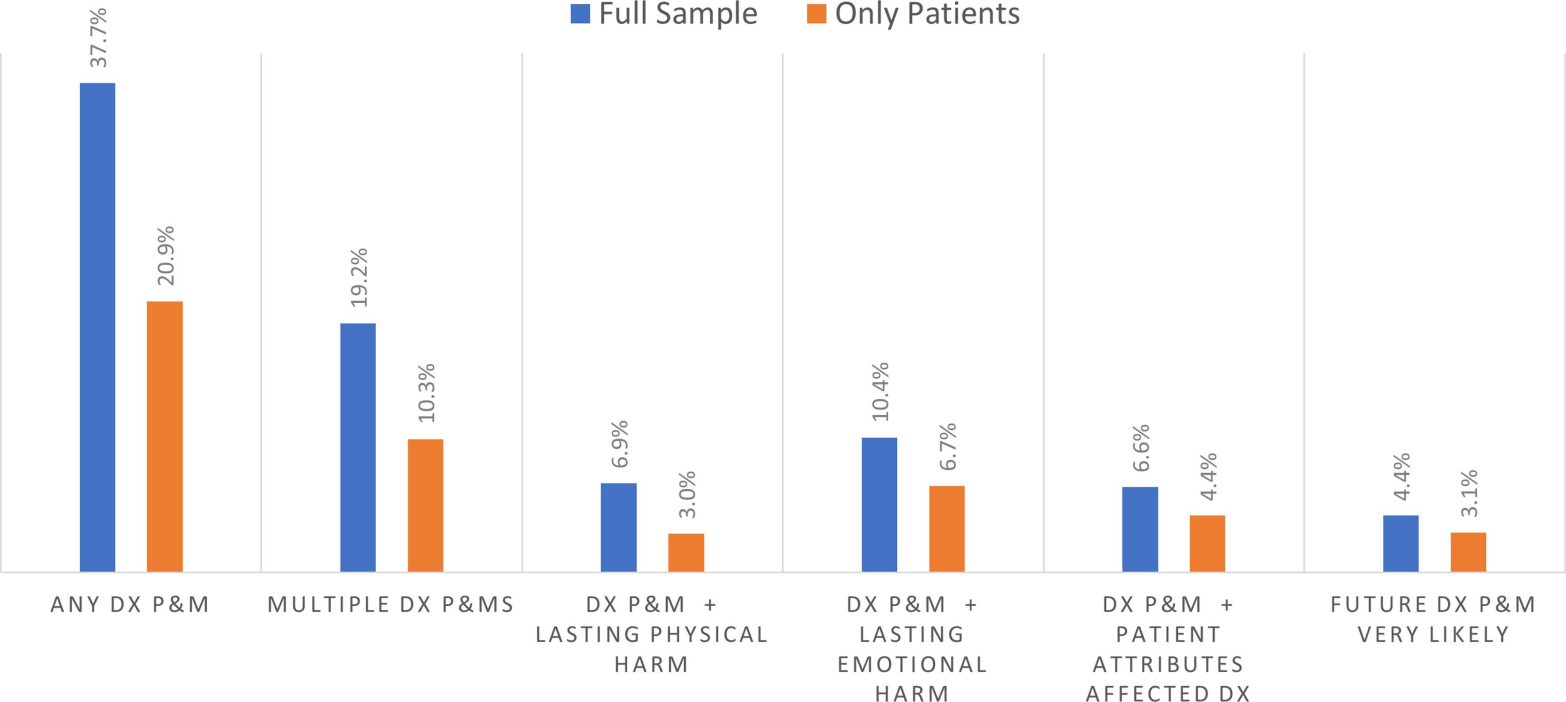
Prevalence of diagnostic problems and mistakes (DX and P&MS) in past 4 years and related outcomes.

Among the 3,995 survey respondents in the household sample, Figure 1 shows that 37.7% reported experiencing at least one diagnostic P&M as a care partner or as a patient in the past 4 years, while 19.2% reported experiencing multiple P&Ms during the same period. Among respondents reporting on their own diagnostic P&Ms (only patients), 20.9% reported experiencing at least one diagnostic P&M in the past 4 years, while 10.3% reported experiencing multiple P&Ms during the same period. The population rate of perceiving personal attributes as impairing diagnosis was 6.6% of household respondents and 4.4% of patients themselves. Figure 1 also shows the prevalence of concerns about having a future diagnostic P&M being very likely in each sample.

### 3.4 Outcomes among respondents who experienced at least one P&M

**Figure 2:**
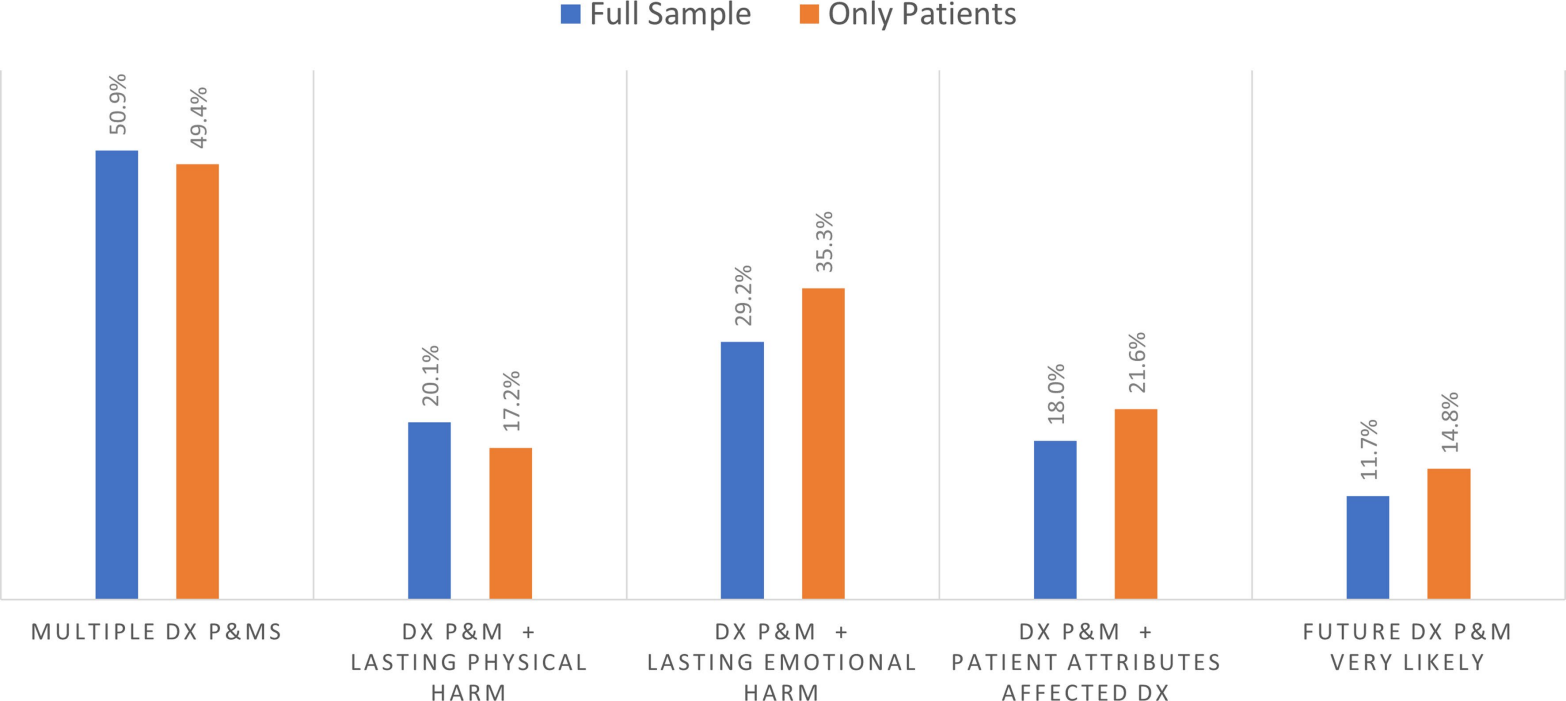
Percentage of outcome for those experiencing at least one diagnostic problem and mistake (DX and P&MS).

Among the 1,506 patient and household care partner respondents reporting on diagnostic P&Ms within their household, Figure 2 shows that about 50% of respondents had experienced multiple P&Ms in the past 4 years. Based on responses for the selected P&M explored in detail in the survey, the longer-term effects resulting from that diagnostic P&M included persisting emotional harm (anxiety) and lasting physical harm. Among household respondents, about 29.2% reported persistent emotional harm, while 20.1% reported persistent physical harm of the person who experienced a P&M (Figure 2). Based on the subsample of patients reporting on themselves, 35.3% reported persisting anxiety, and 17.2% experienced persistent adverse physical effects. Almost 15% of those who experience a P&M thought it was very likely that they would experience another diagnostic P&M in the future.

Almost 22% of patients and 18.0% of the household respondents who reported a diagnostic P&M indicated that a personal attribute had played a role in their problematic diagnostic experience. The personal attributes associated with these pernicious effects sometimes corresponded to sociodemographic categories commonly used in social surveys and identified as sources of disparities in prior studies, but the diagnostic narratives also reported on more finely grained racial, ethnic and cultural identities or other characteristics (e.g., prior diagnosis of mental illness or substance use disorder, large body size). These self-reported attributes and their relationships to diagnostic experiences and outcomes will be examined separately in a forthcoming publication based on the open-ended narrative data.

### 3.5 Sociodemographic predictors of diagnostic P&Ms

Table 3 shows that regression results for both the household level (i.e., respondents reporting either about their own diagnosis or that of another person in their household) and patient subsample (i.e., patients reporting about their own diagnosis) analyses are similar in terms of significant sociodemographic predictors of P&Ms, though some results differ. We report results for each analytic frame separately since strategies for addressing diagnostic inequities may be targeted to individuals or households or both.

**Table 3:** Disparities in prevalence of diagnostic P&Ms within respondent’s household.

Respondent attributes	Frequency of diagnostic P&Ms		Frequency of diagnostic P&Ms with severe impact		Comparison group
	At Least One P&M (Problems + Mistakes)	Multiple P&Ms (in past 4 Years)	Persisting physical harm	Persisting emotional harm	NS (not significant) for *p* > 0.05
	Odds Ratio [prob]	Odds Ratio [prob]	Odds Ratio [prob]	Odds Ratio [prob]	
Patient age (adults)					Age 45–54
18–24 Years Old	2.99 [<0.0001]	2.61 [<0.0001]	1.08 [NS]	1.83 [0.01]	
25–34 Years Old	1.23 [NS]	1.17 [NS]	0.83 [NS]	1.27 [NS]	
35–44 Years Old	1.07 [NS]	1.10 [NS]	1.23 [NS]	1.12 [NS]	
55–64 Years Old	0.88 [NS]	0.71 [NS]	0.71 [NS]	0.72 [NS]	
65–74 Years Old	0.62 [0.001]	0.56 [0.004]	0.55 [NS]	0.48 [0.004]	
75 and Older	0.64 [0.02]	0.46 [0.006]	0.60 [NS]	0.41 [0.01]	
Household income					$100,000 and More
Under $30,000	1.45 [0.005]	1.93 [0.0001]	1.58 [NS]	1.94 [0.002]	
$30,000–$59.999	1.32 [0.02]	1.69 [0.001]	1.75 [0.02]	1.71 [0.006]	
$60.000–$99,999	1.13 [NS]	1.29 [NS]	0.93 [NS]	1.43 [NS]	
Education completed					Some College (inc. AA degree)
Some High School	0.47 [<0.0001]	0.54 [0.005]	0.54 [NS]	0.71 [NS]	
High School Grad	0.72 [0.003]	1.00 [NS]	0.86 [NS]	0.81 [NS]	
College Grad	0.94 [NS]	1.03 [NS]	1.26 [NS]	0.93 [NS]	
Graduate School	1.03 [NS]	1.07 [NS]	0.89 [NS]	0.99 [NS]	
Gender					Cis-Male
Cis-Female	1.25 [0.005]	1.28 [0.02]	1.45 [0.03]	1.36 [0.01]	
Transgender and Gender Independent	5.27 [<0.0001]	2.79 [0.0004]	2.29 [NS]	1.94 [NS]	
Race and ethnicity					White, non-Hispanic
Black, NH	1.03 [NS]	1.00 [NS]	0.64 [NS]	0.61 [NS]	
Hispanic (all races)	1.07 [NS]	1.14 [NS]	0.95 [NS]	0.90 [NS]	
Asian and Pacific Isles, NH	0.73 [NS]	1.23 [NS]	0.46 [NS]	0.61 [NS]	
Other Race, NH	1.03 [NS]	0.29 [NS]	--	0.68 [NS]	
Multiple Races, NH	1.80 [0.007]	1.77 [0.02]	2.17 [0.03]	1.68 [NS]	
Location					Rural/Outside metropolitan
Urban/Metro	0.84 [NS]	0.93 [NS]	0.89 [NS]	1.00 [NS]	
Work status					Not Work-Disabled
Disabled, Unable to Have FT Work	1.94 [<0.0001]	2.28 [<0.0001]	2.76 [0.0001]	2.22 [0.0002]	
Marriage status					Not married
Married	1.03 [NS]	0.90 [NS]	0.89 [NS]	0.97 [NS]	

NH, non-Hispanic; FT, Full-time; NS, not significant.

#### 3.5.1 Household level analysis (full sample of all reported P&Ms)

At the household level, younger patients (18–24 years old) exhibited significantly higher odds of experiencing at least one diagnostic P&M (odds ratio 2.99, *p* < 0.0001) and multiple P&Ms (odds ratio 2.61, *p* < 0.0001) compared to those aged 45–54 years old. Conversely, older patients reported significantly lower odds of experiencing at least one P&M (odds ratio 0.62, *p* = 0.001 for those 65–74 years old and 0.64, *p* = 0.012 for 75 and older) and multiple P&Ms (odds ratio 0.56, *p* = 0.004 for the 65–74 group; and 0.46, *p* = 0.006 for those 75 and older) compared to the reference group (age 45 to 54).

Respondents from households with below average income (under $30,000 and $30,000–$59,999) exhibited significantly higher odds of experiencing at least one diagnostic P&M and multiple P&Ms compared to those from households with incomes of $100,000 and above. The odds ratios followed a consistently inverse gradient with higher and more significant odds for P&Ms (at least one and multiple) as income levels decreased.

Respondents with less education completed, specifically those with only some high school education or high school graduation, exhibited significantly lower odds of experiencing at least one diagnostic P&M compared to those in the reference group of those who had completed some college (odds ratio 0.47, *p* < 0.0001 for some high school; odds ratio 0.72, *p* < 0.003 for high school graduation). Only the lowest educational attainment groups (some high school) reached statistical significance in predictions of multiple P&Ms, and similarly had lower odds compared to the reference group (odds ratio 0.54, *p* = 0.005).

Other significant sociodemographic predictors of increased risk of diagnostic P&Ms are related to gender, race and ethnicity, and disability. Cis-female respondents were significantly more likely to report experiencing any P&M (odds ratio 1.25, *p* = 0.005) and multiple P&Ms (odds ratio 1.28, *p* = 0.02) compared to cis-males. Transgender and gender independent individuals exhibited the highest risks: compared to cis-males (odds ratio 5.27, *p* < 0.0001 for at least one P&M and odds ratio 2.79, *p* = 0.0004 for multiple P&Ms). Disparity predictions for most racial and ethnic groups did not reach statistical significance. However, individuals identifying as multiracial exhibited significantly higher odds of P&Ms compared to non-Hispanic White individuals (odds ratio 1.80, *p* = 0.007 for at least one P&M; and odds ratio 1.77, p = 0.02 for multiple P&Ms). Individuals who identified their work status as disabled (unable to have full-time work) had significantly higher odds of experiencing diagnostic P&Ms compared to those in the reference group (odds ratio 1.94, *p* < 0.0001 for at least one P&M; and odds ratio 2.28, *p* < 0.0001 for multiple P&Ms).

Overall, the findings highlight the presence of disparities in the prevalence of diagnostic P&Ms within households, with significant associations observed for patient age, household income, education, gender, race and ethnicity, and disability status.

#### 3.5.2 Patient-level analysis (subsample reporting about their own diagnosis)

Table 3 also shows the regression analysis for patients reporting about themselves. As with the household analysis, there were no significant differences in the prevalence of diagnostic P&Ms between respondents residing in urban/metro areas compared to those in rural/non-metro areas. Nor were disparities predicted based on marital status (married versus not married).

Both patient age and household income predictions remained similar to the household analysis as expected, given that these sociodemographic characteristics are consistently identified in both samples (Exhibit 2). Patient age was a significant predictor of diagnostic P&Ms, both at least one and multiple P&Ms in the past 4 years. Younger patients (under 25) exhibited significantly higher odds of experiencing at least one diagnostic P&M (odds ratio 3.98, *p* < 0.0001) and multiple P&Ms (odds ratio 3.48, *p* < 0.0001) compared to those aged 45–54. Both these point estimates were higher than in the household analysis. Conversely, older patients (65 and older) had significantly lower odds of experiencing both at least one P&M (odds ratio 0.60, *p* = 0.006 for those 65 to 74; odds ratio 0.58, *p* = 0.03 for those 75 and older) and multiple P&Ms (odds ratio 0.48, *p* = 0.005 for those 65 to 74; odds ratio 0.43, *p* = 0.02 for 75 and older), compared to the reference group.

Respondents with below average incomes again reported more diagnostic P&Ms. Those living in households with the lowest incomes (under $30,000) exhibited significantly higher odds of experiencing P&Ms compared to those from households with incomes of $100,000 and above (odds ratio 1.59, *p* = 0.003 for at least one P&M; odds ratio 2.39, *p* < 0.0001 for multiple P&Ms). For those with household incomes in the next lowest bracket of $30,000–$59,999, respondents reporting about themselves also had significantly greater risk of at least one P&M (odds ratio 1.35, *p* = 0.03) and multiple P&Ms (odds ratio 1.81, *p* = 0.002).

Respondents with lower levels of education, specifically those with only some high school education or high school graduation, exhibited significantly lower odds of experiencing at least one diagnostic P&M compared to the reference group of those who had some college (odds ratio 0.30, *p* < 0.0001 for some high school; odds ratio 0.56, *p* < 0.0001 for high school graduation). Those with some high school also had significantly lower odds of multiple P&Ms (0.37, *p* = 0.003). The differences in point estimates were more pronounced at the patient level compared to the household analysis.

Cis-female respondents had significantly higher odds of experiencing diagnostic P&Ms compared to cis-male respondents (odds ratio 1.2, *p* = 0.04 for at least one P&M; odds ratio 1.35, *p* = 0.02 for multiple P&Ms). Transgender and gender independent individuals also exhibited significantly higher odds of experiencing at least one P&M compared to cis-males (odds ratio 2.04, *p* = 0.05). Although the direction of the effects in the patient analysis were again consistent with the household analysis, the point estimates indicated either the same or less separation from the reference group.

As with the household analysis of racial and ethnic predictors, only individuals identifying as multiracial exhibited significantly higher odds of experiencing P&Ms compared to non-Hispanic White individuals (odds ratio 1.80, *p* = 0.03 for at least one; odds ratio 2.00, p = 0.03 for multiple P&Ms). Point estimates in the household and patient analyses were quite similar.

Disabled (unable to have full-time work) respondents had significantly higher odds of experiencing diagnostic P&Ms compared to the reference group (odds ratio 2.27, *p* < 0.0001 for at least one; odds ratio 2.66, p < 0.0001 for multiple P&Ms).

Overall, the findings highlight the presence of disparities in the prevalence of diagnostic P&Ms affecting respondents’ own healthcare, with significant associations observed for patient age, household income, education completed, gender, race and ethnicity, and disability in both analyses (household and patient only).

### 3.6 Persistent harms from diagnostic P&Ms

Table 4 presents the regression results of sociodemographic predictors of diagnostic P&Ms with severe impacts for both the full household sample and the patient subsample. While the direction of effects of sociodemographic predictors by subgroups is similar in both analyses, there are some predictors that are only statistically significant in one of the analyses.

**Table 4:** Disparities in prevalence of diagnostic P&Ms affecting respondents’ own health care.

Respondent attributes	Frequency of diagnostic P&Ms		Frequency of diagnostic P&Ms with severe impact		Comparison group
	At Least One P&M (Problems + Mistakes)	Multiple P&Ms (In Past 4)	Persisting physical harm	Persisting emotional harm	NS (not significant) for *p* > 0.05
	Odds Ratio [prob]	Odds Ratio [prob]	Odds ratio [prob]	Odds ratio [prob]	
Patient Age (Adults)					Age 45–54
18–24 Years Old	3.98 [<0.0001]	3.48 [<0.0001]	1.91 [NS]	2.26 [0.003]	
25–34 Years Old	1.50 [NS]	1.37 [NS]	0.95 [NS]	1.33 [NS]	
35–44 Years Old	1.24 [NS]	1.23 [NS]	1.30 [NS]	0.95 [NS]	
55–64 Years Old	0.99 [NS]	0.82 [NS]	0.60 [NS]	0.76 [NS]	
65–74 Years Old	0.60 [0.006]	0.48 [0.005]	0.26 [0.006]	0.34 [0.001]	
75 and Older	0.58 [0.03]	0.43 [0.02]	0.31 [NS]	0.31 [0.01]	
Household Income					$100,000 and More
Under $30,000	1.59 [0.003]	2.39 [<0.0001]	1.83 [NS]	2.00 [0.005]	
$30,000–$59.999	1.35 [0.03]	1.81 [0.002]	2.10 [0.03]	1.77 [0.01]	
$60.000–$99,999	1.09 [NS]	1.39 [NS]	1.04 [NS]	1.55 [NS]	
Education Completed					Some College (inc. AA degree)
Some High School	0.30 [<0.0001]	0.37 [0.003]	0.37 [0.05]	0.65 [NS]	
High School Grad	0.56 [<0.0001]	0.75 [NS]	0.61 [NS]	0.66 [0.04]	
College Grad	0.89 [NS]	0.92 [NS]	0.86 [NS]	0.83 [NS]	
Graduate School	1.02 [NS]	1.00 [NS]	0.71 [NS]	1.00 [NS]	
Gender					Cis-Male
Cis-Female	1.22 [0.04]	1.35 [0.02]	1.81 [0.008]	1.55 [0.003]	
Transgender and Gender Independent	2.04 [0.05]	1.87 [NS]	0.89 [NS]	1.68 [NS]	
Race and Ethnicity					White, Non-Hispanic
Black, NH	1.02 [NS]	1.00 [NS]	0.41 [0.03]	0.46 [0.004]	
Hispanic (all races)	0.95 [NS]	1.02 [NS]	0.77 [NS]	0.77 [NS]	
Asian and Pacific Isles, NH	0.95 [NS]	1.66 [NS]	0.74 [NS]	0.58 [NS]	
Other Race, NH	1.15 [NS]	--	--	0.52 [NS]	
Multiple Races, NH	1.80 [0.03]	2.00 [0.03]	1.19 [NS]	1.70 [NS]	
Location					Rural/Outside metropolitan
Urban/Metro	0.92 [NS]	0.92 [NS]	0.79 [NS]	1.06 [NS]	
Work Status					Not Work-Disabled
Disabled, Unable to Have FT Work	2.27 [*p* < 0.0001]	2.66 [<0.0001]	4.11 [<0.0001]	2.55 [0.0002]	
Marriage Status					Not married
Married	1.06 [NS]	0.99 [NS]	0.95 [NS]	0.85 [NS]	

NH, non-Hispanic; FT, Full-time; NS, not significant.

#### 3.6.1 Household level analysis

As shown in the Table 4 household regression results, being cis-female or disabled (unable to have full-time work) was associated with significantly higher odds of both persistent emotional harm (anxiety of the respondent) and persistent physical harm (for the patient) compared to the reference group. For emotional harm, being cis-female increased odds to 1.36 (*p* = 0.01) and being disabled (unable to have full-time work) increased odds to 2.22 (*p* = 0.0002) compared to the reference groups. Being cis-female, disabled or multi-racial was also associated with significantly higher frequencies of persistent physical harm, with odds ratios of 1.45 (*p* = 0.03); 2.76 (*p* = 0.0001); and 2.17 (p = 0.03), respectively.

For the lowest income group (under $30,000), only persisting emotional harms exhibited statistically significant increased likelihood (odds ratio 1.94, *p* = 0.002) compared to the highest household bracket of $100,000 or more. Increased odds of persisting physical and emotional harm were each significant for the $30,000 to 59,999 group (odds ratio 1.75, *p* = 0.02; and odds ratio 1.71, *p* = 0.006, respectively) compared to the reference group. Respondents reporting on patients in the lowest age group (18 through 24 years old) had significantly higher odds of persisting emotional harm (odds ratio 1.83, *p* = 0.01).

Significant predictors of decreased odds of persisting emotional harm after diagnostic P&Ms were seen for older age groups (odds ratio 0.48, *p* = 0.004 for those 65 to 74 years old and 0.41, p = 0.01 for those 75 and older).

#### 3.6.2 Patient level analysis

Similar to the household level regression analysis results, the odds of higher frequencies of persisting harm (physical or emotional) were significant for lower income, cis-female, and disabled (unable to have full-time work) work status (Table 4). The highest and most significant odds of both persisting physical and emotional harms were experienced in the disabled (unable to have full-time work) group compared to the reference group (odds ratio 4.11, *p* < 0.0001 for physical harm; odds ratio 2.55, *p* = 0.0002 for emotional harm). Significantly lower odds for older adults for persisting emotional harms were also present in the patient subsample.

Unlike the household sample, Table 4 shows higher prevalence of persisting physical harms for the 65–74 years old age group compared to those 45 to 54 (odds ratio 0.26, *p* = 0.006). Lower educational attainment significantly predicted reduced odds of physical harm for those with some high school (odds ratio 0.37, *p* = 0.05), and reduced emotional harm for high school graduates (odds ratio 0.66, *p* = 0.04) compared to the reference group.

The significant race and ethnicity predictors were not the same in the patient subsample. Patients who identified as Black, non-Hispanic were also at significantly lower risk of reporting persisting physical harm about oneself (odds ratio 0.41, *p* = 0.03 compared to reference group), as well as persisting emotional harm (odds ratio 0.46, *p* = 0.004). The disparity in prevalence of statistically significant higher odds of persisting physical harm noted for those identifying as multiracial in the household sample was not seen in the patient subsample (odds ratio 1.19 [not significant] versus 2.17, p = 0.03 in the household analysis).

### 3.7 Diagnosis impaired by personal attributes: household and patient-level analyses

Table 5 displays regression results for both analytic frames. For respondents in both samples, the youngest patient group (18–24 years old) was twice as likely to endorse perceiving that a personal attribute impaired diagnosis compared to the reference group (odds ratio 1.84, *p* = 0.04 for household sample; odds ratio 2.02, p = 0.04 for patient sample). The two oldest age categories had significantly lower odds of perceiving that a personal attribute impaired diagnosis with the lowest odds for patients 75 and older.

**Table 5:** Personal attribute perceived as impairing diagnosis during diagnostic P&M.

Respondent attributes	Likelihood of personal attribute effect				Comparison group
	Household analysis		Patients reporting on themselves		NS (not significant) for *p* > 0.05
	Odds Ratio	prob	Odds Ratio	prob	
Patient Age (Adults)					Age 45–54
18–24 Years Old	1.84	0.04	2.02	0.04	
25–34 Years Old	0.99	NS	1.19	NS	
35–44 Years Old	0.83	NS	0.86	NS	
55–64 Years Old	0.69	NS	0.73	NS	
65–74 Years Old	0.42	0.005	0.38	0.008	
75 and Older	0.44	0.05	0.28	0.02	
Household Income					$100,000 and More
Under $30,000	1.83	0.02	1.97	0.03	
$30,000–$59.999	1.79	0.02	1.69	NS	
$60.000–$99,999	1.29	NS	1.33	NS	
Education Completed					Some College (inc. AA degree)
Some High School	0.22	0.002	0.10	0.002	
High School Grad	0.65	0.05	0.41	0.001	
College Grad	1.28	NS	1.05	NS	
Graduate School	1.15	NS	1.20	NS	
Gender					Cis-Male
Cis-Female	2.12	<0.0001	2.48	<0.0001	
Transgender and Gender Independent	5.36	<0.0001	5.64	<0.0001	
Race and Ethnicity					White, Non-Hispanic
Black, NH	0.83	NS	0.78	NS	
Hispanic (all races)	0.90	NS	0.83	NS	
Asian and Pacific Isles, NH	0.62	NS	0.22	NS	
Other Race, NH	0.80	NS	1.07	NS	
Multiple Races, NH	1.77	NS	1.40	NS	
Location					Rural/Outside metropolitan
Urban/Metro	0.85	NS	1.02	NS	
Work Status					Not Work-Disabled
Disabled, Unable to Have FT Work	2.48	0.001	2.31	0.009	
Marriage Status					Not Married
Married	0.84	NS	0.87	NS	

NH, non-Hispanic; FT, Full-time; NS, not significant.

In both the household and patient samples, higher odds of experiencing an impaired diagnosis based on personal attributes were significantly predicted at almost twice the odds for the lowest income brackets (under $30,000) as well as for the next lowest bracket ($30,000–$59,999) for the household sample compared to reference group ($100,000 and more). Lower levels of educational attainment significantly predicted much lower likelihood of perceived personal attribute effect, with the lowest odds reported by patients themselves with some high school (odds ratio 0.10, *p* = 0.002) compared to the reference group (some college education).

Higher rates of perceiving a personal attribute impaired diagnosis were predicted for cis-female gender and transgender and gender independent groups compared to the cis-male group in both samples (odds ratio 2.48, *p* < 0.0001 and odds ratio of 5.64, p < 0.0001, respectively). Inability to work due to a disability was also consistently associated with an elevated rate of reporting that diagnosis had been disrupted by a personal attribute.

### 3.8 Expectations for future diagnostic risks

Table 6 illustrates several disparities in expectations for future diagnostic risks among respondents, as assessed by the likelihood of the respondent anticipating a future diagnostic P&M occurring when receiving health care. We report both household and patient-level analyses in the table side-by-side.

**Table 6:** Disparities in expectations for future diagnostic risks.

Respondent attributes	Likelihood of future diagnostic P&M				Comparison group
	Household analysis		Patients reporting on themselves		NS (not significant) for *p* > 0.05
	Odds Ratio	prob	Odds Ratio	prob	
Patient Age (Adults)					Age 45–54
18–24 Years Old	1.11	NS	0.90	NS	
25–34 Years Old	0.87	NS	0.73	NS	
35–44 Years Old	1.15	NS	0.93	NS	
55–64 Years Old	0.84	NS	0.79	NS	
65–74 Years Old	0.57	0.02	0.49	0.02	
75 and Older	0.57	NS	0.42	0.03	
Household Income					$100,000 and More
Under $30,000	1.18	NS	1.19	NS	
$30,000–$59.999	1.26	NS	1.34	NS	
$60.000–$99,999	1.08	NS	1.26	NS	
Education Completed					Some College (inc. AA degree)
Some High School	0.99	NS	1.16	NS	
High School Grad	1.04	NS	1.06	NS	
College Grad	1.08	NS	0.93	NS	
Graduate School	1.21	NS	1.06	NS	
Gender					Cis-Male
Cis-Female	1.30	0.03	1.60	0.002	
Transgender and Gender Independent	1.33	NS	3.34	0.01	
Race and Ethnicity					White, Non-Hispanic
Black, NH	0.76	NS	0.77	NS	
Hispanic (all races)	0.73	NS	0.87	NS	
Asian and Pacific Isles, NH	0.98	NS	1.27	NS	
Other Race, NH	0.88	NS	0.76	NS	
Multiple Races, NH	1.57	NS	1.25	NS	
Location					Rural/Outside metropolitan
Urban/Metro	1.37	0.05	1.10	NS	
Work Status					Not Work-Disabled
Disabled, Unable to Have FT Work	1.24	NS	1.19	NS	
Marriage Status					Not Married
Married	0.81	NS	0.82	NS	

NH, non-Hispanic; FT, Full-time; NS, not significant.

Older age groups had significantly lower odds of expecting a future diagnostic P&M compared to the reference group (odds ratio 0.49, *p* = 0.02 for 65–74; odds ratio 0.42, *p* = 0.03 for 75 and older for patients reporting on themselves). A similar pattern holds for the household level data, which include expectations reported by care partners. Within this sample, only the 65 to 74 group had statistically significantly lower odds of concern (odds ratio 0.57, p = 0.02).

In comparison to cis-males, cis-females and transgender and gender independent individuals who had experienced a diagnostic P&M had significantly higher odds of expecting future diagnostic P&Ms (odds ratio 1.60, *p* = 0.0002 for cis-females; odds ratio 3.34, *p* = 0.01 for transgender and gender independent in the household analysis). In the household sample, the cis-female group, but not the transgender and gender independent group, had statistically significant higher odds of concern.

In both cases, the gender-related differences in future risk were consistent with the differences in P&M and harm experiences reported in Tables 3, 4. By contrast, households in urban/metro areas reported statistically higher odds of concern about future diagnostic P&Ms (odds ratio 1.37, *p* = 0.05). though these elevated risk perceptions were not matched by any comparable geographic differences in the experience of diagnostic P&Ms or harms.

## 4 Discussion

Our study aimed to fill several noteworthy gaps in the literature on diagnostic safety. First, it enriches our understanding of patient-reported diagnostic P&Ms by augmenting earlier findings estimating the national prevalence of harmful diagnostic events by incorporating multiple P&Ms, harmful consequences, and P&Ms attributable to differential treatment based on identified personal attributes. These new findings offer valuable insights into the prevalence of diagnostic breakdowns and their distribution across various sociodemographic groups, shedding light on disparities that may exist in healthcare experiences and outcomes related to diagnosis. The consistency of findings across multiple outcomes increases our confidence that these at-risk groups merit greater attention and protections.

Second, we successfully demonstrated the feasibility of obtaining patient-reported data from a national sample to better understand diagnostic P&Ms and their sociodemographic predictors. This includes responses from population subgroups that have historically had limited opportunities to voice problems and mistakes during their diagnostic experiences. And it includes data from narrative accounts that illuminate interactions within the diagnostic process in ways not previously visible to researchers.

Our analyses revealed several types of noteworthy findings that we group into three clusters. The first set involves results that are broadly consistent with findings from past studies, but which highlight nuances not identified in previous research. The second set of findings illuminate new sources of disparities for which we have not previously had reliable national estimates of magnitude, and fresh aspects or perspectives that more fundamentally alter how we should think about or address diagnostic inequities. The final cluster is in some ways the most generative, raising a variety of questions or puzzles that merit attention in future research.

In discussing our findings, we utilize definitions of health equity, diagnostic equity, health disparities and diagnostic disparities summarized in the public briefing book for the National Academies Workshop: “Advancing Equity in Diagnostic Excellence to Reduce Health Disparities.” (46) Specifically, health equity is “the state in which everyone has a fair and just opportunity to attain their highest level of health.” (47), while diagnostic equity is defined as “providing everyone with a fair and just chance of receiving a timely, accurate diagnosis to lead to appropriate interventions and health benefits, regardless of personal characteristics.” (5, 20, 48) Similarly, health disparities are defined as “preventable differences in the burden of disease, injury, violence, or opportunities,” (49) and “diagnostic disparities occur when diagnostic errors are experienced at disproportionate rates by certain patient subgroups based, for example, on patients’ age, sex/gender, or race/ethnicity.” (50) In our study, diagnostic disparities reflect experiences of problems and/or mistakes during a patient’s diagnostic journey (diagnostic P&Ms), which may or may not be classified as diagnostic errors from a clinical point of view.

### 4.1 Better understanding previously documented diagnostic disparities

Our findings are largely consistent with those in the literature identifying elevated risk of diagnostic difficulties for young adults, cis-women, those living in low-income households and people with disabilities. In each case, however, the findings reported above highlight some implications that have been overlooked or downplayed in past research.

Younger adult patients, particularly those under 25 years old, experienced significantly higher rates of diagnostic P&Ms compared to their older counterparts. While prior studies have pointed to risks of delayed or missed diagnosis for younger people for specific clinical conditions (e.g., stroke, young adult cancers) (51, 52) and patient or clinician perceptions of the patient “being too young” for the diagnosis they ultimately received (41, 53–55), the population-based estimates of double to triple the chance of diagnostic P&Ms for this younger age group in our multivariate analysis suggests a need for bringing greater attention to both clinical and non-clinical contributors of this elevated risk.

Previous research has documented gender-related biases in diagnosis related to cis-women compared to cis-men, most commonly in terms of clinicians’ dismissal of symptoms reported by patients (56, 57). These prior findings are echoed most strongly in our findings reported in Table 5, which highlights gendered differences that respondents observed in their interactions during diagnosis. But the elevated rates of diagnostic risk for cis-female respondents are also evident for persistent harms in the aftermath of diagnostic breakdowns. This could reflect a second-stage of dismissal, if cis-women’s reports of symptoms related to diagnostic P&M itself are also taken less seriously than are comparable reports from cis-men.

Lower household income also emerged as a significant predictor of higher prevalence of diagnostic P&Ms, with the lowest income group facing the highest risks. This association is consistent with multiple qualitative and quantitative studies that single-out economic disadvantage as a predictor of diagnostic breakdowns (12, 14). But previous research involves samples too small to distinguish the scope of these financial risks. Our findings suggest that the scope is quite extensive—with all Americans living in households with below-average income experiencing elevated risks of diagnostic P&Ms. Developing effective interventions to mitigate the impact of financial barriers on diagnostic accuracy and timeliness likely will depend on close attention to dynamics both inside and outside of the medical system, as well to difficulties at the boundary of these two terrains that people must navigate as they become patients during a diagnostic process (5).

Previous research has also identified a variety of ways in which physical disabilities impair testing and other aspects of the diagnostic process (58). Because our analyses relied on the identification of disability through work status (disabled and unable to have full-time work), it suggests an alternative or additional pathway for increased diagnostic risks in part due to different levels of connection to medical care or less extensive support with clinical issues from workplace human resource departments. Because disability can affect employment, social status, and sources of insurance in this way, our findings underscore the importance of recognizing and addressing the unique needs of disabled individuals within healthcare systems that go beyond clinical interactions, emphasizing the imperative for tailored interventions and supports. Interventions could also be developed based on analogous efforts in other targeted areas such as food insecurity for those who have disability (whether related to work status or not) to apply best practices for accessibility, universal design, and maximize input from the disability rights community (59, 60).

### 4.2 Newly identified aspects of diagnostic disparities

Our analysis identified other sociodemographic predictors of diagnostic P&Ms, affecting individuals who self-identify as transgender and gender independent, as well as those who identify as multiracial. Both groups were associated with substantially increased risk of experiencing diagnostic P&Ms and associated harms. But neither has received much attention in past research, despite their strikingly elevated risks.

In both cases, this situation reflects a common reluctance among researchers to report statistical results for subgroups that represent a relatively small portion of the American public. In fact, many studies explicitly suppress findings for subsamples that fall below an arbitrary size threshold (56). Consequently, smaller groups like those identifying as transgender or gender independent or those identifying as multiracial (both representing 2–3% of the American public) do not get reported in results, no matter how large the cross-group differences are in diagnostic or other health-related experiences.

This practice rests on inadequate statistical reasoning. To be sure, if sample sizes are small, the standard errors on the regression coefficients get inflated, and even large cross group differences may sometimes be statistically insignificant. (Note, for example, the nonsignificant but large odds-ratios on persisting harms for the transgender and gender independent respondents in Table 3. Or observe the persisting emotional harms for multiracial respondents in that same table.) But when comparisons remain statistically significant despite the small sample sizes, they often illuminate strikingly pronounced disparities, as can be observed for the transgender and gender independent respondents in Table 5. These should not be ignored.

A second set of new findings reflect subgroups of respondents who report substantially *fewer* diagnostic P&Ms or harms than the average patient. Here again, this is evident for two sets of respondents: those with more limited education and those over the age of 65. Consider first individuals with lower levels of education, particularly those with some high school education or having completed high school. Past statistical studies of diagnostic P&Ms have typically included either measures of education *or* measures of household income, but not both. Because our findings reveal that low-income is associated with increased risks, but lower education is associated with lower reported diagnostic P&Ms, failing to include both variables means that the two relationships would statistically cancel each other out, making it appear that lower socioeconomic status has no strong relationship to diagnostic outcomes at all.

Our finding, by contrast, thus opens space to hypothesize about why these offsetting associations exist. Perhaps individuals with lower education levels face fewer diagnostic problems compared to those with higher levels of education, though that seems unlikely. Alternatively, it may be that they are significantly less likely to report these effects. The challenges of adjusting to the complex terminology and terrain of health care among individuals with limited education, especially in the diagnostic stage of care, may make it harder to recognize and report diagnostic P&Ms (14, 28), resulting in underestimation of their prevalence as well as their impact. Alternatively, individuals with lower education levels may have developed lower expectations for healthcare, potentially leading them to be less likely to report deficits in care or attribute harms to diagnostic P&Ms. Further research is needed to explore the complex interplay between education level, healthcare expectations, health literacy, and diagnostic outcomes to inform strategies for improving healthcare quality and equity across diverse socioeconomic backgrounds.

As reported in our findings above, older adults, aged 65 and above, consistently demonstrated reduced odds of diagnostic P&Ms and harms. This might seem surprising, given the multiple comorbidities and polypharmacy that increase as people age, increasing the exposure to health care and any iatrogenic risks. However, one study in the UK found that older adults were more likely to have both higher expectations and be more satisfied with their care compared to younger patients (61). As expectations are socially constructed to a large degree, one’s generational context (e.g., life as a “baby boomer”), as well as one’s prior experiences within a given country’s health system, could be relevant and potentially produce different patterns of expectations and reporting by patients and their care partners about health care experiences. Alternatively, the more stable and health-promoting coverage of the Medicare program may facilitate more regular visits to clinicians and thus more timely diagnoses among older Americans.

Finally, our study is the first to identify disparities in expectations regarding future diagnostic risks. Certain subgroups, including cis-females, and transgender and gender independent individuals particularly express heightened concerns. These findings emphasize the need for proactive measures to address patient anxieties about future care, improve communication, and address trust breaches between patients and healthcare providers, including interventions aimed at acknowledgement and repair. Additionally, more directed attention to how patients and their care partners reflect on their diagnostic P&M experiences and outcomes could deepen considerations about different ways that concerns about their future care could manifest (30).

### 4.3 Further puzzles and priorities for future research

Our findings illuminate a number of patterns among experiences and expectations regarding diagnostic disparities that merit additional attention from scholars and additional prioritization among funders of medical and health services research. We describe here five puzzling results that seem particularly deserving of future scrutiny.

First, there are some noteworthy differences in the relationship between experiences with diagnostic P&Ms (Tables 3–5) and expectations regarding future risks (Table 6). Gendered differences in risk of P&Ms and harms are matched by elevated concerns about future risk among cis-women and transgender or gender independent respondents. But other subgroups experiencing equally elevated P&Ms—such as respondents with disabilities who are unable to have full-time work or those living in households with below-average income—do not appear to translate those experiences into elevated perceptions of future risk. Similar inconsistencies emerge for those reporting fewer diagnostic risks. Older Americans’ perceptions of below-average diagnostic P&Ms and harms are matched by their expectations of lower future risks. But a comparable consistency of reduced experiences and expectations does *not* carry over to respondents with limited education. Better understanding the origins of these inconsistencies might offer useful insights into how people understand or interpret their past diagnostic experiences, their future expectations, or both.

Second, as noted above, the association between household income and diagnostic risks extends over a surprisingly large portion of the public. Authors of past studies have inferred that there might be a relationship between Medicaid coverage, reduced reimbursement rates for clinician visits, limited time spent in diagnosis, and consequently, elevated risk of diagnostic breakdowns (7, 12). But Medicaid coverage for adults is limited almost exclusively to those in the bottom quartile of the income distribution. Since elevated diagnostic risks and harms extends to the bottom *two* quartiles, some other causal or associative pathway must be in play. Research is needed to identify what that entails.

Third, our findings suggest that there is close congruence between subgroups that report identified diagnostic risk (individual or multiple P&Ms), diagnostic harms (persisting physical or emotional distress) and perceptions that patients were diagnosed differently and sub-optimally based on some identifiable personal attribute. What sort of interactions lead patients or care partners to make these attributions? And how are they able to discern this differential diagnostic process, when they are only observing their own or a care partner’s diagnosis and not the experiences of other patients they do not know? These questions merit additional study.

Fourth, how might the perception that patients have been treated differently during diagnosis because of some personal attribute alter patients’ (or care partners’) longer-term relationships with individual clinicians or with the healthcare system as a whole? The excerpts in Table 2 highlight these perceptions, such as the respondent who stated: “And being a person with a disability you are treated as though you cannot possibly be competent to make your own decisions. I am confident that if these factors were not present, things would have gone differently.” Is perceived discrimination, in particular, corrosive to trust in medical care or in health care professionals or both? Are there ways in which more positive expectations might be restored, despite a perception of past discrimination or other issues raised by these respondents? Here again, additional research is needed to address these questions.

Finally, contrary to expectation, a set of null findings is particularly vexing. In our multivariate analyses, except for predictors related to the multiracial group, other race and ethnicity groups did not emerge as a significant predictor of elevated diagnostic P&Ms or associated outcomes. These findings may reflect the complex and intersectional nature of healthcare disparities, where the influence of race and ethnicity on diagnostic outcomes may be mediated by other factors such as socioeconomic status. However, prior literature suggests grave inequities among racial and ethnic minorities arising from structural barriers, implicit bias, overt racism, and differential access to high-quality care (21, 62). It is vital to highlight that the statistical meaning of a null finding is not proof of no effect. Future research with larger samples will allow interaction analysis with race and ethnicity categories to further explore associations with diagnostic P&Ms and harms. At the same time, it is also possible that other explanations (e.g., concerns and resulting hesitations about reporting problems related to health care) deserve more attention in future studies of diagnostic P&Ms. For example, a scoping review found evidence of underreporting by clinicians of patient safety events for Black patients compared to White patients in voluntary reporting systems, which could correspond to biases in information supplied directly to Black patients and their care partners about what went wrong in their care (63).

### 4.4 The broader context of inequities based on other U.S.-based surveys

That diagnostic shortfalls perceived by patients and their families are unevenly distributed in the U.S. should, in itself, be unsurprising. Past surveys have long documented persisting inequities in Americans’ reported economic insecurity (64), social anxieties (65, 66), and stigma related to various health conditions (67). Surveys of Americans’ experiences within health care have similarly documented multiple inequities, including those related to gender identity (13), race/ethnicity (68, 69), disabling conditions (70), socio-economic status (71), and immigration status (72).

Although the existence of unequal experiences has been extensively documented and is generally understood by most Americans (73), less widely recognized is an important corollary: that the magnitude and specific patterns of inequities often varies across outcomes in some crucial ways. This was evident in some of our findings. Although those living in low-income households are generally at risk for elevated level of adverse events while receiving healthcare, these risks have in many past studies been concentrated in the lowest quartile of the income distribution. By contrast, findings reported here suggest that the risk of diagnostic mistakes and/or problems is elevated among all households with below-average incomes. Apart from revealing a much wider population at risk, it is these discrepant patterns that offer clues to the origins of certain types of inequitable outcomes.

During the past several decades, patient experience surveys have been widely deployed throughout the U.S. healthcare system, perhaps most impactfully as a means for incentivizing hospitals to promote patient-centered practices (74). Most of these surveys are designed to generate feedback on events that are more prevalent than safety shortfalls, so they have provided relatively little guidance on either the frequency or the inequities in safety experiences, including those occurring during diagnosis, often over time and across multiple settings.

### 4.5 The role of health delivery systems for the future of diagnostic equity

While the survey results quantify the magnitude of disparities in diagnosis and unveil potential subgroups experiencing diagnostic-related inequities, the implications for the health delivery system may appear hazy. However, when viewed through the looking glass of potentially different perceptions on the concept of diagnosis—those of diverse patients and clinicians—the need to look anew from all angles merits discussion. When interpreting data derived from patient and care partner experiences, a common critique is that their perceptions about diagnosis may differ from clinical experts, and that the latter somehow trumps the former. Such debates limit subsequent steps to those aimed at sorting out differences between patient and clinician perceptions, as opposed to seizing the opportunity to gain unique insights from diverse and nationally representative samples of the public through surveys such as the one analyzed in this paper. Health delivery systems are in a pivotal position to implement complementary approaches, including stratification of patient level data based on sociodemographic characteristics to evaluate safety and quality disparities. Such stratified analysis would be enriched by expanding patient level data to include questions about experience of the diagnostic process and outcomes. Health delivery system engagement in pursuing incorporation of such data gathering from their patients and neighborhood citizens would facilitate in-depth and local efforts to integrate the complementary expertise of patients, care partners, clinicians and public health officials.

### 4.6 The role of future research in advancing diagnostic equity

Future research is also pivotal to making progress toward diagnostic equity. First, expanding beyond the illustrative excerpts provided in this paper would include a rigorous qualitative examination of the narratives that accompany the quantitative results from our survey. Second, to the extent that health systems might respond to these results, we anticipate that research that aims to connect diagnostically-focused survey results to currently collected information from health systems about patient satisfaction and experience would be valuable.

### 4.7 Limitations of the existing study

Despite the valuable insights gained from our study, several limitations must be acknowledged. First, the reliance on self-reported data may introduce social desirability bias, potentially leading to underreporting or overreporting of diagnostic P&Ms and associated outcomes. Past research suggests that patients and care partners will have difficulty separating out diagnostic mistakes that were preventable from adverse events that were not (32). However, understanding both types of diagnostic breakdown is still important and may yield persisting harms, including reducing trust in future diagnostic reliability or safety. Moreover, self-reported outcomes can also identify diagnostic breakdowns that are in clinicians’ blindspots, thereby enhancing diagnostic safety (40).

Second, the cross-sectional design of the study precludes establishing causality or temporal relationships between sociodemographic factors and diagnostic outcomes. This limits our ability to infer causation, since statistical associations may embody forms of reverse causality. For example, the odds-ratios identified in the regression models connecting disability status with elevated P&Ms may reflect P&Ms causing work disabilities, rather than patients with disabilities facing greater vulnerability to diagnostic P&Ms.

Third, while efforts were made to ensure the representativeness of the sample to the general U.S. population, inherent biases in survey participation and sampling may have influenced the findings, limiting the generalizability of the results. The most pronounced bias was induced by our reliance on an internet panel for recruiting respondents involves literacy, since people who regularly complete surveys on-line clearly have a reading capacity that is not universal among the American public. That may lead our results to understate the impact of low literacy and limited education on diagnostic outcomes.

Fourth, the use of broad categories for sociodemographic variables based on the questions pre-determined for the nationally representative panel used in this study may overlook the heterogeneity within the available subgroups (e.g., for race and ethnicity, work-related disability status) and obscure important nuances in healthcare experiences and outcomes.

Fifth, while our sample is the largest yet for patient-reported diagnostic P&Ms, it is not large enough for thorough interaction analyses to explore the numerous intersectional predictors worthy of exploration. This is particularly consequential if patients’ perception of stigma linked to some personal attribute or aspect of their medical history might become a more pronounced barrier to effective diagnosis for patients who have multiple stigmatizing conditions.

Sixth, in choosing to focus on diagnostic P&Ms from the unique voice and lived experiences of patients and their household care partners, we do not make any claims about clinical adjudication of these reports or potential classifications as diagnostic errors from a medical perspective. Even if diagnostic P&M prevalence and associated harm estimates, along with the sociodemographic patterns revealed in this study, differ to some extent from clinically adjudicated diagnostic errors or breakdowns, this study provides a public health foundation for making progress on diagnostic equity by centering on lived experiences of the public.

Finally, while our study provides valuable insights into demographic disparities in diagnostic outcomes (diagnostic P&Ms and associated harms), the complexity of healthcare disparities warrants further investigation into the underlying mechanisms driving these disparities. Future research employing longitudinal designs and drawing more heavily on qualitative methodologies than did this study may provide a more comprehensive understanding of the factors contributing to diagnostic disparities and inform targeted interventions to improve healthcare equity.

## 5 Conclusion

In our assessment, this study provides valuable insights into the prevalence and sociodemographic correlates of diagnostic P&Ms, shedding light on the complex interplay between patient characteristics and healthcare experiences. The findings reveal significant sociodemographic disparities in diagnostic P&Ms, with younger patients, those with lower household income, cis-women, transgender and gender independent individuals, those with individuals with multiracial identities and those who are disabled (unable to have full-time work) being particularly vulnerable.

Moreover, disparities were observed in not only the frequency of diagnostic P&Ms, but also the impact of diagnostic P&Ms, with low-income individuals, cis-females and disabled individuals experiencing higher rates of persistent emotional and physical harm. Younger patients also experience higher rates of persisting emotional harm. These findings underscore the need for targeted interventions to address systemic biases and promote equitable access to high-quality healthcare for all individuals, regardless of their demographic characteristics.

Overall, the findings from this study contribute to a deeper understanding of healthcare disparities and underscore the importance of addressing systemic biases in healthcare delivery. By identifying vulnerable populations and disparities in healthcare experiences, policymakers, healthcare providers, and researchers can develop targeted interventions to improve diagnostic accuracy, enhance patient-provider communication, and promote healthcare equity. Ultimately, addressing demographic disparities in diagnostic P&Ms is essential for achieving the goal of providing high-quality, patient-centered care to all individuals, regardless of their sociodemographic characteristics.

Future research should further explore the underlying mechanisms driving these disparities and evaluate the effectiveness of interventions aimed at mitigating diagnostic P&Ms and errors across diverse sociodemographic groups. By better understanding the origins and implications of disparate diagnostic experiences, we should be able to more effectively identify actionable strategies for reducing the prevalence and impact of diagnostic breakdowns in the future, thereby relieving the burdens on those subgroups who are disproportionately experiencing them now.

## Data Availability

The raw data supporting the conclusions of this article will be made available by the authors, without undue reservation.
