# Supplementary material for: Exploring sociodemographic disparities in diagnostic problems and mistakes in the quest for diagnostic equity: insights from a national survey of patient experiences
Source: Front Public Health. 2025 Feb 13;13:1444005. doi: 10.3389/fpubh.2025.1444005 (PMC11865055; doi:10.3389/fpubh.2025.1444005)
Supplement: Supplementary file 1 [file Data_Sheet_1.docx]

Background: As part of building a platform for epidemiological research on diagnostic errors and problems that centers on patients and equity, we developed a novel survey (referred to as NEP-DE) to explore the prevalence and harm consequences of diagnostic problems or mistakes (referred to here as “diagnostic P&Ms”).

Data Source: We conducted a population-based survey of a nationally representative sample in 2022-2023, drawn from the NORC AmeriSpeak® probability-based panel that provides sample coverage of approximately 97% of the U.S. household population. Eligible participants were patients or household care partners ages 18 or older. The survey response modes available were Computer-Assisted Web Interviewing (CAWI) and Computer-Assisted Telephone Interviewing (CATI).

Sociodemographic Data: NORC collects the following sociodemographic data from each participant: age (18-24, ten year groups, 75+), income (Under $30,000, $30,000-$59,999, $60,000-$99,999, $100,000 and Above), education (less than high school, high school degree, some college, college degree, and postgraduate study/degree), gender identity (cis-male, cis-female, transgender and gender independent), race and ethnicity (non-Hispanic White, Black, Asian, Other Race, Multiple Races; and Hispanic), urban/rural residence, disability related to full-time work status (yes, no), and marital status.

Narrative Elicitation Protocol Diagnostic P&Ms Survey(NEP-DE):

The survey includes 35 questions, a subset of which were used in this paper’s quantitative analysis. The questions utilized for the quantitative analysis of outcomes appear in Exhibit 1 of the paper. This Appendix provides further context: the key components of the survey, and the survey’s open-ended questions used to assemble illustrative excerpts of respondent responses related to outcomes assessed.

*Initial Questions: Whether the respondent had experienced one or more diagnostic P&Ms*

The survey first asks if any household member has experienced a diagnostic P&M within the last four years. For those who answered affirmatively, a follow-up question determined whether multiple events had occurred. If the respondent has their own personal experience, they are instructed to detail the most significant event related to their own healthcare. In the absence of personal experience, they are asked to report on the most notable event involving a household member, prioritizing events with substantial impact.

*Subsequent Questions about the most impactful diagnostic P&M:*

Section 1 of the survey includes close-ended questions *About the diagnostic P&M* (e.g., who it happened to; when it happened) and open-ended questions (see below).

Section 2 questions focus on *Discovery of and response to the diagnostic P&M* (e.g., how the respondent first came to realize there was a problem getting a clear and accurate diagnosis; whether an apology about the diagnostic problem was received)*.*

Section 3 questions relate to the *Impact of the diagnostic P&M* (e.g. whether the diagnostic P&M caused persisting physical or emotional harm; anticipated likelihood of a future diagnostic P&M).

*The questions below were used in the accompanying paper to assemble illustrative excerpts, and for classifications related to the outcome for personal attributes contributing to impaired diagnosis.*

**Ten Open-ended Questions in Narrative Elicitation Sequence (excerpted from Section 1 script)**

Now we have some questions to learn more about this diagnostic mistake or problem and the impact that it had. Hearing about this situation in your own words can help doctors and other clinicians avoid similar problems in the future and better help their patients deal with diagnostic problems that still happen.

Hearing about these experiences in your own words helps doctors and other clinicians do a better job diagnosing health problems and communicating about them to patients and families. We value your contribution here.

In your own words, tell us more about the diagnostic mistake or problem that happened. Please describe what happened, who was involved, and what might have led up to the problem.

[LARGE TEXTBOX]

Now, please describe how well talking, listening and other forms of communicating went between [INSERT DOV_PRONOUN1] and [INSERT DOV_PRONOUN4] providers throughout the diagnostic process.

[LARGE TEXTBOX]

After [INSERT DOV_PRONOUN5] realized that there was a problem with the diagnosis, what, if anything, did the doctors and other clinicians do or say that made things better? This could include things that improved [DOV_PRONOUN2] health, [DOV_PRONOUN2] medical care, or how [DOV_PRONOUN5] felt about the diagnostic experiences.

[LARGE TEXTBOX]

After [INSERT DOV_PRONOUN5] realized that there was a problem with the diagnosis, what, if anything, did the doctors and other clinicians do that made things worse? This could include anything that negatively impacted [INSERT DOV_PRONOUN2] health, [INSERT DOV_PRONOUN2] medical care, or how [INSERT DOV_PRONOUN5] felt about the diagnostic experiences?

[LARGE TEXTBOX]

After [INSERT DOV_PRONOUN5] realized there was a diagnostic problem, what, if anything, do [INSERT DOV_PRONOUN5] wish had been done by doctors, clinicians, or others in the healthcare system to improve the situation?

[LARGE TEXTBOX]

Please explain how, if at all, this diagnostic mistake or problem affected [INSERT DOV_PRONOUN2] life and [INSERT DOV_PRONOUN2] medical care in the months immediately following when [INSERT DOV_PRONOUN5] realized that there was a problem. How were [INSERT DOV_PRONOUN5] feeling about these experiences?

[LARGE TEXTBOX]

How, if at all, did the experience of this diagnostic mistake or problem affect the ways in which [INSERT DOV_PRONOUN5] currently use the health care system? Did it impact when and how [INSERT DOV_PRONOUN5] seek medical care? How [INSERT DOV_PRONOUN5] interact with doctors and other clinicians?

[LARGE TEXTBOX]

The next questions ask you to look back on all the experiences dating back to the start of the search for a diagnosis for these health problems.

Hearing about these experiences in your own words helps doctors and other clinicians do a better job diagnosing health problems and communicating about them to patients and families. We value your contribution here.

Are there things you understand now about [INSERT DOV_PRONOUN2] diagnosis or the diagnostic process that you wish you had known sooner? Please explain.

[LARGE TEXTBOX]

Did [INSERT DOV_PRONOUN5] have a clinician or other person who [INSERT DOV_PRONOUN5] felt was a reliable source of guidance and support during the diagnostic process? If so, how did [INSERT DOV_PRONOUN5] come to rely on this person and how did this person help?

[LARGE TEXTBOX]

Sometimes aspects of people’s background, culture, identity or health needs make their diagnostic experiences better or worse. How, if at all, do you think these factors impacted [INSERT DOV_PRONOUN2] diagnostic experience?
